# RKIP: A Pivotal Gene Product in the Pathogenesis of Cancer

**DOI:** 10.3390/cancers14246092

**Published:** 2022-12-11

**Authors:** Benjamin Bonavida

**Affiliations:** Department of Microbiology, Immunology and Molecular Genetics, David Geffen School of Medicine, College of Life Sciences, UCLA, Los Angeles, CA 90095, USA; bbonavida@mednet.ucla.edu

Raf kinase inhibitor protein (RKIP), previously known as a phosphatidylethanolamine-binding protein (PEBP), was cloned by Yeung et al. [1]and found to be an inhibitor of the RAF/MEK/ERK signaling pathway. RKIP was originally found in bovine brain and is a small cytosolic protein that is highly conserved across species such as monkeys, chickens, rats, and humans. Since this early discovery, several reports in the literature demonstrated the pleiotropic activities of RKIP in the pathogenesis of cancer and other non-cancerous tissues. In particular, RKIP is known to play a role in the MAPK and NF-*κ*B signaling pathways, but a loss of RKIP can lead to the progression of cancer and other diseases.

In several cancers, the expression of RKIP is low; thus, the MAPK and NF- *κ*B signaling pathways are constitutively activated, maintaining tumor growth, metastasis, and resistance to both chemotherapeutic drugs and immunotherapies.

This Special Issue in *Cancers*, titled *“RKIP: A Pivotal Gene Product in the Pathogenesis of Cancer”*, compiles several of the most recent research investigations attributed to RKIP roles in various cancers and its central regulation of the intracellular signaling pathways and their cross-talks defining the unique phenotypes of cancers and, further, RKIP’s role as a significant prognostic biomarker and therapeutic target. This special issue consists of eight review chapters, each of which discusses novel findings and altogether constitutes a significant reference on the rapidly evolving RKIP field.

Below, I will briefly highlight the main findings and their significance that were discussed in each chapter.

Lorenz and Rosner, in “*Harnessing RKIP to combat heart disease and cancer* [2], reviewed two leading diseases to high morbidity and mortality and addressed the role of RKIP in the regulation of these two diseases. In its unphosphorylated form, RKIP acts as a metastatic suppressor, while in its phosphorylated form, it acts as a protector of heart failure. These investigators examined how to treat cancer by inhibiting the Raf/ERK1/2 pathway without the consequence of stimulating cardiotoxicity. They discussed two approaches to achieve this goal: the use of a partial mimic of non-phosphorytable RKIP is an approach to treat metastatic cancer and leaving RKK1/2 activity largely intact, thus avoiding cardiotoxicity. The second approach is to selectively target pERK with a peptide that inhibits ERK dimerization, which does not interfere with ERK1/2 activation and its anti-apoptotic activity in cardiomyocytes.

Bach et al., in “*A negative role for RKIP in breast cancer immune response* “ [3], investigated whether the inhibition of tumor progression and metastasis by RKIP also involves other targets such as the immune response. They discuss the reported role of RKIP in the regulation of inflammation initiated by both innate and adaptive immune responses. The underlying mechanism of the RKIP-mediated enhancement of T cell-mediated inflammatory response is not known via the production of IFN- γ. Vu et al. [3] examined the role of RKIP on immune response genes in breast cancer. They showed that knocking down RKIP increased the expression of enriched genes that are vital for type 1 and 2 interferon-mediated inflammatory responses in breast cancer cells. Thus, they provided evidence that RKIP is indeed an immune modulator in breast cancer.

Giovanni et al., in “*A stochastic binary model for the regulation of gene expression to investigate responses to gene therapy*” [4], researched treatment responses with a switching target using a two-state stochastic model for gene expression. They used the *RKIP* gene and two non-specific drugs that are known to change RKIP levels in cancer cells as a model. These various experimental designs helped clarify how environmental information may be processed by cells via investigations on how the regulation of the RKIP gene expression affects its downstream kinases.

Cessna et al. in “ *The role of RKIP in the regulation of EMT on the tumor microenvironment”* [5], reviewed and discussed whether the RKIP-mediated suppression of EMT and metastasis underlies its role in the negative regulation of the mesenchymal gene products associated with EMT and the positive regulation of the epithelial gene products. An analysis of various signaling pathways established cross-talks between the negative and positive gene regulations mediated by RKIP. The findings showed that the tumor suppressor RKIP regulated the expression of mesenchymal gene products, such as Snail, vimentin, E-cadherin, laminin, and EPCAM. This suggested that agents that can selectively induce RKIP expressionsin cancer cells should result in reversing the cancer cell phenotype and the inhibition of cell proliferation, invasion, metastasis, and resistance relative to both chemotherapeutic drugs and immunotherapy.

Papale et al., in “*Understanding mechanisms of RKIP regulation to improve the development of new diagnostic tools”* [6], investigated the transcriptional, post-transcriptional, and post-translational regulation of both the expression and activation of RKIP in the appropriate tumors. They discuss the role of RKIP phosphorylation as a potential positive or negative prognostic factor depending on the type of neoplasm and the microenvironment of such a neoplasm. They analyzed various cancers, including lung, colon, breast, myeloid neoplasms and multiple myeloma, melanoma, and clear cells renal cell carcinoma. In addition, they also reviewed RKIP in biological fluids. These various analyses were performed in an effort to clarify the potential of RKIP expression as an accurate biomarker for the diagnosis and prognosis of cancers.

Vivarelli et al., in “*Computational analyses of YY1 and its target RKIP reveal their diagnostic and prognostic roles in lung cancer* “ [7], investigated the current challenges in various treatment options for lethal lung cancer. Treated patients often experience relapses and generalized metastases. Furthermore, many patients do not respond to these treatment strategies. Thus, there is an urgent need to discover new biomarkers for the prognosis and diagnosis of lung cancer patients. As the expression of Yin Yang 1 (YY1) is overexpressed in lung cancer and the expression of the tumor suppressor Raf Kinase Inhibitory Protein (RKIP) is underexpressed in lung cancer, Vivarelli et al. performed computational analyses and found that YY1 negatively regulated RKIP expression in lung cancer. They demonstrated that such analyses and observed differences correlated positively with a diagnostic significance. In addition, they also validated that YY1 and RKIP represented prognostic biomarkers. They also speculated that both YY1 and RKIP are also therapeutic targets in lung cancer.

Ahmed et al., in “*A functional network model of the metastasis suppressor PEB1/RKIP and its regulators in breast cancer cells* “ [8], investigated, via text mining and a literature survey, means for extracting interactions between metastasis suppressors and their regulators. Using a breast cancer cell line, MCF7, they demonstrated that two regulatory mechanisms are involved in the regulation of RKIP. One mechanism invokes previously reported RELA (transcription factor p65) and SNAIL1, and the second mechanism involved the estrogen receptor (ESR1), which induces RKIP via kinase NME1. They proposed that that these two mechanisms are likely targets for therapeutic interventions against metastatic breast cancer.

Dong et al., in “*Insights of RKIP-derived suppression of prostate cancer*” [9], reviewed the tumor suppressive activities of RKIP in prostate cancer. Prostate cancer (PC) remains a major cause of cancer death in men, and no effective treatments have been developed for metastatic PC. Therefore, there is an urgent need to discover a new therapy to intervene in metastatic PC to prevent death. Whereas the metastatic process in PC is complex and involves various factors, RKIP plays an important role as it is downregulated in PC. The overexpression of RKIP in PC results in several manifestations, such as the inhibition of proliferation, the inhibition of invasion, and the reversal of the resistance to cytotoxic therapeutics. However, the underlying molecular and genetic mechanisms involved in RKIP-mediated roles in PC have not as yet been accomplished, and the authors discuss various approaches for such investigations.

Clearly, as discussed above, these various contributions provided up-to date investigations on the important tumor suppressor, RKIP, in various human cancers. They included novel investigations as well as reviews that altogether provide comprehensive information on the most recent developments on RKIP in various cancers and also in non-cancer diseases. The important role of RKIP in both prognosis and diagnosis was highlighted and targeting RKIP is a novel method for therapeutically inhibiting tumor progression and metastasis as well as reverse drug resistance.

The Section Editor, Benjamin Bonavida, acknowledges all of the contributing authors for their efforts and their significant investigations on RKIP and he also acknowledges all of the staff of the publisher of *Cancers*.

Of interest, a follow up of these contributions above can be followed in a forthcoming International Conference titled “*Prognostic and Therapeutic Implications of RKIP and YY1 in Cancer, Diabetes and Cardiovascular Diseases*” which will be held in Catania, Sicily, Italy, 8–11 March 2023.

## References

[1] Yeung K., Seitz T., Li S., Janosch P., McFerran B., Kaiser C., Fee F., Katsanakis K.D., Rose D.W., Mischak H. (1999). Suppression of Raf-1 kinase activity and MAP kinase signalling by RKIP. Nature.

[2] Lorenz K., Rosner M.R. (2022). Harnessing RKIP to Combat Heart Disease and Cancer. Cancers.

[3] Bach V.N., Ding J., Yeung M., Conrad T., Odeh H.N., Cubberly P., Figy C., Ding H.-F., Trumbly R., Yeung K.C. (2022). A Negative Regulatory Role for RKIP in Breast Cancer Immune Response. Cancers.

[4] Giovanini G., Barros L.R.C., Gama L.R., Tortelli T.C., Ramos A.F. (2022). A Stochastic Binary Model for the Regulation of Gene Expression to Investigate Responses to Gene Therapy. Cancers.

[5] Cessna H., Baritaki S., Zaravinos A., Bonavida B. (2022). The Role of RKIP in the Regulation of EMT in the Tumor Microenvironment. Cancers.

[6] Papale M., Netti G.S., Stallone G., Ranieri E. (2022). Understanding Mechanisms of RKIP Regulation to Improve the Development of New Diagnostic Tools. Cancers.

[7] Vivarelli S., Falzone L., Grillo C.M., Bonavida B., Crimi C., La Mantia I., Libra M. (2022). Computational Analyses of YY1 and Its Target RKIP Reveal Their Diagnostic and Prognostic Roles in Lung Cancer. Cancers.

[8] Ahmed M., Lai T.H., Kim W., Kim D.R. (2021). A Functional Network Model of the Metastasis Suppressor PEBP1/RKIP and Its Regulators in Breast Cancer Cells. Cancers.

[9] Dong Y., Lin X., Kapoor A., Gu Y., Xu H., Major P., Tang D. (2021). Insights of RKIP-Derived Suppression of Prostate Cancer. Cancers.

